# Supplementary material for: Surgical referral systems in low- and middle-income countries: A review of the evidence
Source: PLoS One. 2019 Sep 27;14(9):e0223328. doi: 10.1371/journal.pone.0223328 (PMC6764741; doi:10.1371/journal.pone.0223328)
Supplement: S2 Table — (DOCX) [file pone.0223328.s002.docx]

**S2 Table: Search string**

The search string was originally tested in the MEDLINE database and then adapted and run in EMBASE and Global Health databases.

MEDLINE

| ((Hospital Referral[Title/Abstract]) OR Hospital Referrals[Title/Abstract]) OR ((referral*[Title/Abstract]) AND consultation*[Title/Abstract])) OR ((Referral and Consultation[MeSH Terms])) |
| --- |
| AND |
| ((surgical procedure[Title/Abstract] OR surgical procedures[Title/Abstract]) OR operative procedure[Title/Abstract] OR operative procedures[Title/Abstract] OR "Surgical Procedures, Operative"[Mesh] OR "Digestive System Surgical Procedures"[Mesh] OR "Elective Surgical Procedures"[Mesh] OR "Obstetric Surgical Procedures"[Mesh] OR "Orthopedic Procedures"[Mesh]) |
| AND |
| ((Deprived Countries[tw] OR Deprived Population[tw] OR Deprived Populations[tw] OR Developing Countries[tw] OR Developing Country[tw] OR Developing Economies[tw] OR Developing Economy[tw] OR Developing Nation[tw] OR Developing Nations[tw] OR Developing Population[tw] OR Developing Populations[tw] OR Developing World[tw] OR LAMI Countries[tw] OR LAMI Country[tw] OR Less Developed Countries[tw] OR Less Developed Country[tw] OR Less Developed Economies[tw] OR Less Developed Nation[tw] OR Less Developed Nations[tw] OR Less Developed World[tw] OR Lesser Developed Countries[tw] OR Lesser Developed Nations[tw] OR LMIC[tw] OR LMICS[tw] OR Low GDP[tw] OR Low GNP[tw] OR Low Gross Domestic[tw] OR Low Gross National[tw] OR Low Income Countries[tw] OR Low Income Country[tw] OR Low Income Economies[tw] OR Low Income Economy[tw] OR Low Income Nations[tw] OR Low Income Population[tw] OR Low Income Populations[tw] OR Lower GDP[tw] OR lower gross domestic[tw] OR Lower Income Countries[tw] OR Lower Income Country[tw] OR Lower Income Nations[tw] OR Lower Income Population[tw] OR Lower Income Populations[tw] OR Middle Income Countries[tw] OR Middle Income Country[tw] OR Middle Income Economies[tw] OR Middle Income Nation[tw] OR Middle Income Nations[tw] OR Middle Income Population[tw] OR Middle Income Populations[tw] OR Poor Countries[tw] OR Poor Country[tw] OR Poor Economies[tw] OR Poor Economy[tw] OR Poor Nation[tw] OR Poor Nations[tw] OR Poor Population[tw] OR Poor Populations[tw] OR poor world[tw] OR Poorer Countries[tw] OR Poorer Economies[tw] OR Poorer Economy[tw] OR Poorer Nations[tw] OR Poorer Population[tw] OR Poorer Populations[tw] OR Third World[tw] OR Transitional Countries[tw] OR Transitional Country[tw] OR Transitional Economies[tw] OR Transitional Economy[tw] OR Under Developed Countries[tw] OR Under Developed Country[tw] OR under developed nations[tw] OR Under Developed World[tw] OR Under Served Population[tw] OR Under Served Populations[tw] OR Underdeveloped Countries[tw] OR Underdeveloped Country[tw] OR underdeveloped economies[tw] OR underdeveloped nations[tw] OR underdeveloped population[tw] OR Underdeveloped World[tw] OR Underserved Countries[tw] OR Underserved Nations[tw] OR Underserved Population[tw] OR Underserved Populations[tw]) OR (Afghanistan[tw] OR Albania[tw] OR Algeria[tw] OR "American Samoa"[tw] OR Angola[tw] OR Armenia[tw] OR Azerbaijan[tw] OR Bangladesh[tw] OR Belarus[tw] OR Byelarus[tw] OR Belorussia[tw] OR Belize[tw] OR Benin[tw] OR Bhutan[tw] OR Bolivia[tw] OR Bosnia[tw] OR Botswana[tw] OR Brazil[tw] OR Bulgaria[tw] OR Burma[tw] OR "Burkina Faso"[tw] OR Burundi[tw] OR "Cabo Verde"[tw] OR "Cape verde"[tw] OR Cambodia[tw] OR Cameroon[tw] OR "Central African Republic"[tw] OR Chad[tw] OR China[tw] OR Colombia[tw] OR Comoros[tw] OR Comores[tw] OR Comoro[tw] OR Congo[tw] OR "Costa Rica"[tw] OR "Cote d'Ivoire"[tw] OR Cuba[tw] OR Djibouti[tw] OR Dominica[tw] OR "Dominican Republic"[tw] OR Ecuador[tw] OR Egypt[tw] OR "El Salvador"[tw] OR Eritrea[tw] OR Ethiopia[tw] OR Fiji[tw] OR Gabon[tw] OR Gambia[tw] OR Gaza[tw] OR "Georgia Republic"[tw] OR Georgian[tw] OR Ghana[tw] OR Grenada[tw] OR Grenadines[tw] OR Guatemala[tw] OR Guinea[tw] OR Guyana[tw] OR Haiti[tw] OR Herzegovina[tw] OR Hercegovina[tw] OR Honduras[tw] OR India[tw] OR Indonesia[tw] OR Iran[tw] OR Iraq[tw] OR Jamaica[tw] OR Jordan[tw] OR Kazakhstan[tw] OR Kenya[tw] OR Kiribati[tw] OR Korea[tw] OR Kosovo[tw] OR Kyrgyz[tw] OR Kirghizia[tw] OR Kirghiz[tw] OR Kirgizstan[tw] OR Kyrgyzstan[tw] OR "Lao PDR"[tw] OR Laos[tw] OR Lebanon[tw] OR Lesotho[tw] OR Liberia[tw] OR Libya[tw] OR Macedonia[tw] OR Madagascar[tw] OR Malawi[tw] OR Malay[tw] OR Malaya[tw] OR Malaysia[tw] OR Maldives[tw] OR Mali[tw] OR "Marshall Islands"[tw] OR Mauritania[tw] OR Mauritius[tw] OR Mexico[tw] OR Micronesia[tw] OR Moldova[tw] OR Mongolia[tw] OR Montenegro[tw] OR Morocco[tw] OR Mozambique[tw] OR Myanmar[tw] OR Namibia[tw] OR Nepal[tw] OR Nicaragua[tw] OR Niger[tw] OR Nigeria[tw] OR Pakistan[tw] OR Palau[tw] OR Panama[tw] OR "Papua New Guinea"[tw] OR Paraguay[tw] OR Peru[tw] OR Philippines[tw] OR Phillippines[tw] OR Philipines[tw] OR Phillipines[tw] OR Principe[tw] OR Romania[tw] OR Rwanda[tw] OR Ruanda[tw] OR Samoa[tw] OR "Sao Tome"[tw] OR Senegal[tw] OR Serbia[tw] OR "Sierra Leone"[tw] OR "Solomon Islands"[tw] OR Somalia[tw] OR "South Africa"[tw] OR "South Sudan"[tw] OR "Sri Lanka"[tw] OR "St Lucia"[tw] OR "St Vincent"[tw] OR Sudan[tw] OR Suriname[tw] OR Swaziland[tw] OR Syria[tw] OR "Syrian Arab Republic"[tw] OR Tajikistan[tw] OR Tadzhikistan[tw] OR Tadjikistan[tw] OR Tadzhik[tw] OR Tanzania[tw] OR Thailand[tw] OR Timor[tw] OR Togo[tw] OR Tonga[tw] OR Tunisia[tw] OR Turkey[tw] OR Turkmen[tw] OR Turkmenistan[tw] OR Tuvalu[tw] OR Uganda[tw] OR Ukraine[tw] OR Uzbek[tw] OR Uzbekistan[tw] OR Vanuatu[tw] OR Vietnam[tw] OR "West Bank"[tw] OR Yemen[tw] OR Zambia[tw] OR Zimbabwe[tw])) |

EMBASE

| (hospital AND referral* OR 'patient referral'/exp OR (referral AND consultation)) AND (surgical AND procedure* OR (operative AND procedure*)) |
| --- |
| AND |
| ('deprived countries' OR (deprived AND countries) OR 'deprived population' OR (deprived AND ('population'/exp OR population)) OR 'deprived populations' OR (deprived AND populations) OR 'developing countries'/exp OR 'developing countries' OR (developing AND countries) OR 'developing country'/exp OR 'developing country' OR (developing AND country) OR 'developing economies' OR (developing AND economies) OR 'developing economy' OR (developing AND ('economy'/exp OR economy)) OR 'developing nation' OR (developing AND nation) OR 'developing nations' OR (developing AND nations) OR 'developing population' OR (developing AND ('population'/exp OR population)) OR 'developing populations' OR (developing AND populations) OR 'developing world' OR (developing AND ('world'/exp OR world)) OR 'lami countries' OR (lami AND countries) OR 'lami country' OR (lami AND country) OR 'less developed countries' OR (less AND developed AND countries) OR 'less developed country'/exp OR 'less developed country' OR (less AND developed AND country) OR 'less developed economies' OR (less AND developed AND economies) OR 'less developed nation' OR (less AND developed AND nation) OR 'less developed nations' OR (less AND developed AND nations) OR 'less developed world' OR (less AND developed AND ('world'/exp OR world)) OR 'lesser developed countries' OR (lesser AND developed AND countries) OR 'lesser developed nations' OR (lesser AND developed AND nations) OR lmic OR lmics OR 'low gdp' OR (low AND ('gdp'/exp OR gdp)) OR 'low gnp' OR (low AND gnp) OR 'low gross domestic' OR (low AND gross AND domestic) OR 'low gross national' OR (low AND gross AND national) OR 'low income countries' OR (low AND ('income'/exp OR income) AND countries) OR 'low income country'/exp OR 'low income country' OR (low AND ('income'/exp OR income) AND country) OR 'low income economies' OR (low AND ('income'/exp OR income) AND economies) OR 'low income economy'/exp OR 'low income economy' OR (low AND ('income'/exp OR income) AND ('economy'/exp OR economy)) OR 'low income nations' OR (low AND ('income'/exp OR income) AND nations) OR 'low income population'/exp OR 'low income population' OR (low AND ('income'/exp OR income) AND ('population'/exp OR population)) OR 'low income populations' OR (low AND ('income'/exp OR income) AND populations) OR 'lower gdp' OR (lower AND ('gdp'/exp OR gdp)) OR 'lower gross domestic' OR (lower AND gross AND domestic) OR 'lower income countries' OR (lower AND ('income'/exp OR income) AND countries) OR 'lower income country' OR (lower AND ('income'/exp OR income) AND country) OR 'lower income nations' OR (lower AND ('income'/exp OR income) AND nations) OR 'lower income population' OR (lower AND ('income'/exp OR income) AND ('population'/exp OR population)) OR 'lower income populations' OR (lower AND ('income'/exp OR income) AND populations) OR 'middle income countries' OR (middle AND ('income'/exp OR income) AND countries) OR 'middle income country'/exp OR 'middle income country' OR (middle AND ('income'/exp OR income) AND country) OR 'middle income economies' OR (middle AND ('income'/exp OR income) AND economies) OR 'middle income nation' OR (middle AND ('income'/exp OR income) AND nation) OR 'middle income nations' OR (middle AND ('income'/exp OR income) AND nations) OR 'middle income population'/exp OR 'middle income population' OR (middle AND ('income'/exp OR income) AND ('population'/exp OR population)) OR 'middle income populations' OR (middle AND ('income'/exp OR income) AND populations) OR 'poor countries' OR (poor AND countries) OR 'poor country' OR (poor AND country) OR 'poor economies' OR (poor AND economies) OR 'poor economy' OR (poor AND ('economy'/exp OR economy)) OR 'poor nation' OR (poor AND nation) OR 'poor nations' OR (poor AND nations) OR 'poor population' OR (poor AND ('population'/exp OR population)) OR 'poor populations' OR (poor AND populations) OR 'poor world' OR (poor AND ('world'/exp OR world)) OR 'poorer countries' OR (poorer AND countries) OR 'poorer economies' OR (poorer AND economies) OR 'poorer economy' OR (poorer AND ('economy'/exp OR economy)) OR 'poorer nations' OR (poorer AND nations) OR 'poorer population' OR (poorer AND ('population'/exp OR population)) OR 'poorer populations' OR (poorer AND populations) OR 'third world' OR (third AND ('world'/exp OR world)) OR 'transitional countries' OR (transitional AND countries) OR 'transitional country' OR (transitional AND country) OR 'transitional economies' OR (transitional AND economies) OR 'transitional economy' OR (transitional AND ('economy'/exp OR economy)) OR 'under developed countries' OR (under AND developed AND countries) OR 'under developed country'/exp OR 'under developed country' OR (under AND developed AND country) OR 'under developed nations' OR (under AND developed AND nations) OR 'under developed world' OR (under AND developed AND ('world'/exp OR world)) OR 'under served population' OR (under AND served AND ('population'/exp OR population)) OR 'under served populations' OR (under AND served AND populations) OR 'underdeveloped countries'/exp OR 'underdeveloped countries' OR (underdeveloped AND countries) OR 'underdeveloped country'/exp OR 'underdeveloped country' OR (underdeveloped AND country) OR 'underdeveloped economies' OR (underdeveloped AND economies) OR 'underdeveloped nations' OR (underdeveloped AND nations) OR 'underdeveloped population' OR (underdeveloped AND ('population'/exp OR population)) OR 'underdeveloped world' OR (underdeveloped AND ('world'/exp OR world)) OR 'underserved countries' OR (underserved AND countries) OR 'underserved nations' OR (underserved AND nations) OR 'underserved population' OR (underserved AND ('population'/exp OR population)) OR 'underserved populations' OR (underserved AND populations) OR 'afghanistan'/exp OR afghanistan OR 'albania'/exp OR albania OR 'algeria'/exp OR algeria OR 'american samoa'/exp OR 'american samoa' OR (('american'/exp OR american) AND ('samoa'/exp OR samoa)) OR 'angola'/exp OR angola OR 'armenia'/exp OR armenia OR 'azerbaijan'/exp OR azerbaijan OR 'bangladesh'/exp OR bangladesh OR 'belarus'/exp OR belarus OR 'byelarus'/exp OR byelarus OR 'belorussia'/exp OR belorussia OR 'belize'/exp OR belize OR 'benin'/exp OR benin OR 'bhutan'/exp OR bhutan OR 'bolivia'/exp OR bolivia OR bosnia OR 'botswana'/exp OR botswana OR 'brazil'/exp OR brazil OR 'bulgaria'/exp OR bulgaria OR 'burma'/exp OR burma OR 'burkina faso'/exp OR 'burkina faso' OR (burkina AND faso) OR 'burundi'/exp OR burundi OR 'cabo verde'/exp OR 'cabo verde' OR (cabo AND verde) OR 'cape verde'/exp OR 'cape verde' OR (cape AND verde) OR 'cambodia'/exp OR cambodia OR 'cameroon'/exp OR cameroon OR 'central african republic'/exp OR 'central african republic' OR (('central'/exp OR central) AND ('african'/exp OR african) AND republic) OR 'chad'/exp OR chad OR 'china'/exp OR china OR 'colombia'/exp OR colombia OR 'comoros'/exp OR comoros OR comores OR comoro OR 'congo'/exp OR congo OR 'costa rica'/exp OR 'costa rica' OR (('costa'/exp OR costa) AND rica) OR 'cote divoire' OR (cote AND divoire) OR 'cuba'/exp OR cuba OR 'djibouti'/exp OR djibouti OR 'dominica'/exp OR dominica OR 'dominican republic'/exp OR 'dominican republic' OR (dominican AND republic) OR 'ecuador'/exp OR ecuador OR 'egypt'/exp OR egypt OR 'el salvador'/exp OR 'el salvador' OR (el AND ('salvador'/exp OR salvador)) OR 'eritrea'/exp OR eritrea OR 'ethiopia'/exp OR ethiopia OR 'fiji'/exp OR fiji OR 'gabon'/exp OR gabon OR 'gambia'/exp OR gambia OR gaza OR 'georgia republic' OR (('georgia'/exp OR georgia) AND republic) OR georgian OR 'ghana'/exp OR ghana OR 'grenada'/exp OR grenada OR grenadines OR 'guatemala'/exp OR guatemala OR 'guinea'/exp OR guinea OR 'guyana'/exp OR guyana OR 'haiti'/exp OR haiti OR herzegovina OR hercegovina OR 'honduras'/exp OR honduras OR 'india'/exp OR india OR 'indonesia'/exp OR indonesia OR 'iran'/exp OR iran OR 'iraq'/exp OR iraq OR 'jamaica'/exp OR jamaica OR 'jordan'/exp OR jordan OR 'kazakhstan'/exp OR kazakhstan OR 'kenya'/exp OR kenya OR 'kiribati'/exp OR kiribati OR 'korea'/exp OR korea OR 'kosovo'/exp OR kosovo OR kyrgyz OR 'kirghizia'/exp OR kirghizia OR kirghiz OR kirgizstan OR 'kyrgyzstan'/exp OR kyrgyzstan OR 'lao pdr' OR (lao AND pdr) OR 'laos'/exp OR laos OR 'lebanon'/exp OR lebanon OR 'lesotho'/exp OR lesotho OR 'liberia'/exp OR liberia OR 'libya'/exp OR libya OR macedonia OR 'madagascar'/exp OR madagascar OR 'malawi'/exp OR malawi OR 'malay'/exp OR malay OR 'malaya'/exp OR malaya OR 'malaysia'/exp OR malaysia OR 'maldives'/exp OR maldives OR 'mali'/exp OR mali OR 'marshall islands'/exp OR 'marshall islands' OR (marshall AND ('islands'/exp OR islands)) OR 'mauritania'/exp OR mauritania OR 'mauritius'/exp OR mauritius OR 'mexico'/exp OR mexico OR 'micronesia'/exp OR micronesia OR 'moldova'/exp OR moldova OR 'mongolia'/exp OR mongolia OR 'montenegro'/exp OR montenegro OR 'morocco'/exp OR morocco OR 'mozambique'/exp OR mozambique OR 'myanmar'/exp OR myanmar OR 'namibia'/exp OR namibia OR 'nepal'/exp OR nepal OR 'nicaragua'/exp OR nicaragua OR 'niger'/exp OR niger OR 'nigeria'/exp OR nigeria OR 'pakistan'/exp OR pakistan OR 'palau'/exp OR palau OR 'panama'/exp OR panama OR 'papua new guinea'/exp OR 'papua new guinea' OR (('papua'/exp OR papua) AND new AND ('guinea'/exp OR guinea)) OR 'paraguay'/exp OR paraguay OR 'peru'/exp OR peru OR 'philippines'/exp OR philippines OR phillippines OR philipines OR phillipines OR principe OR 'romania'/exp OR romania OR 'rwanda'/exp OR rwanda OR ruanda OR 'samoa'/exp OR samoa OR 'sao tome' OR (sao AND tome) OR 'senegal'/exp OR senegal OR 'serbia'/exp OR serbia OR 'sierra leone'/exp OR 'sierra leone' OR (sierra AND leone) OR 'solomon islands'/exp OR 'solomon islands' OR (solomon AND ('islands'/exp OR islands)) OR 'somalia'/exp OR somalia OR 'south africa'/exp OR 'south africa' OR (south AND ('africa'/exp OR africa)) OR 'south sudan'/exp OR 'south sudan' OR (south AND ('sudan'/exp OR sudan)) OR 'sri lanka'/exp OR 'sri lanka' OR (sri AND lanka) OR 'st lucia'/exp OR 'st lucia' OR (('st'/exp OR st) AND lucia) OR 'st vincent' OR (('st'/exp OR st) AND vincent) OR 'sudan'/exp OR sudan OR 'suriname'/exp OR suriname OR 'swaziland'/exp OR swaziland OR 'syria'/exp OR syria OR 'syrian arab republic'/exp OR 'syrian arab republic' OR (('syrian'/exp OR syrian) AND ('arab'/exp OR arab) AND republic) OR 'tajikistan'/exp OR tajikistan OR 'tadzhikistan'/exp OR tadzhikistan OR tadjikistan OR tadzhik OR 'tanzania'/exp OR tanzania OR 'thailand'/exp OR thailand OR 'timor'/exp OR timor OR 'togo'/exp OR togo OR 'tonga'/exp OR tonga OR 'tunisia'/exp OR tunisia OR 'turkey'/exp OR turkey OR turkmen OR 'turkmenistan'/exp OR turkmenistan OR 'tuvalu'/exp OR tuvalu OR 'uganda'/exp OR uganda OR 'ukraine'/exp OR ukraine OR uzbek OR 'uzbekistan'/exp OR uzbekistan OR 'vanuatu'/exp OR vanuatu OR 'vietnam'/exp OR vietnam OR 'west bank' OR (west AND bank) OR 'yemen'/exp OR yemen OR 'zambia'/exp OR zambia OR 'zimbabwe'/exp OR zimbabwe) |

GLOBAL HEALTH

| ((referral or referrals or referring) and (surgery or surgeries or surgical)).ab. |
| --- |
| AND |
| ('developing countries' or 'low income countries' or 'middle income countries' or afghanistan or albania or algeria or 'american samoa' or angola or armenia or azerbaijan or bangladesh or belarus or belize or benin or bhutan or bolivia or bosnia or botswana or brazil or bulgaria or burma or 'burkina faso' or burundi or 'cabo verde' or cambodia or cameroon or 'central african republic' or chad or china or colombia or comoros or congo or 'costa rica' or 'cote divoire' or cuba or djibouti or dominica or 'dominican republic' or ecuador or egypt or 'el salvador' or eritrea or ethiopia or fiji or gabon or gambia or gaza or georgia or ghana or grenada or guatemala or guinea or guyana or haiti or herzegovina or honduras or india or indonesia or iran or iraq or jamaica or jordan or kazakhstan or kenya or kiribati or korea or kosovo or kirgizstan or laos or lebanon or lesotho or liberia or libya or macedonia or madagascar or malawi or malaysia or maldives or mali or 'marshall islands' or mauritania or mauritius or mexico or micronesia or moldova or mongolia or montenegro or morocco or mozambique or myanmar or namibia or nepal or nicaragua or niger or nigeria or pakistan or palau or panama or 'papua new guinea' or paraguay or peru or philippines or principe or romania or rwanda or samoa or 'sao tome' or senegal or serbia or 'sierra leone' or 'solomon islands' or somalia or 'south africa' or 'south sudan' or 'sri lanka' or 'st lucia' or 'st vincent' or sudan or suriname or swaziland or syria or tajikistan or tanzania or thailand or timor or togo tunisia or turkey or turkmenistan or tuvalu or uganda or ukraine or uzbekistan or vanuatu or vietnam or 'west bank' or yemen or zambia or zimbabwe).gl. |
