# Supplementary material for: Surgical referral systems in low- and middle-income countries: A review of the evidence
Source: PLoS One. 2019 Sep 27;14(9):e0223328. doi: 10.1371/journal.pone.0223328 (PMC6764741; doi:10.1371/journal.pone.0223328)
Supplement: S3 Table — (DOCX) [file pone.0223328.s003.docx]

**S3 Table. Characteristics of studies included in the review**

| **Authors / year** | **Country** | **Population** | **Setting** | **Joanna Briggs Institute’s level of evidence** | **Study description** | **Study rigour** |
| --- | --- | --- | --- | --- | --- | --- |
| Albutt et al 2018 | Uganda | 35 interviews with hospital staff (clinicians and administrators) | 1 national referral hospital, 8 general district hospitals, 8 regional hospitals | Level 4.b | Qualitative study using semi-structured interviews to understand challenges in the provision of surgical care country-wide in Uganda. | This study reports on a country-wide surgical capacity assessment. The referral system is discussed, but not main focus. |
| Crandon et al 2008 | Jamaica | 122 trauma patients transferred to the hospital for admission or investigations | 1 tertiary referral hospital | Level 4.c | Quantitative descriptive study assessing inter-hospital transfer of trauma patient based on prospective examination of patients clinical status, review of pre-transfer communication, assessment of vehicle, transfer personnel and equipment. Six months of data. | Sound study. However, only 1 investigator involved in the independent assessment, which may increase risk of bias. |
| den Hollander et al 2014 | South Africa | 462 patients referred for operative management of burn injuries | 1 tertiary referral hospital | Level 4.c | Descriptive study of epidemiology and patterns of burn referrals based on a retrospective patients charts review. Two years of data. | Sound study but no limitations mentioned by authors. Methodology for determining appropriateness of referrals only based on type of burn. Analysis of other potential underlying factors leading to the referral not included. |
| Goodman et al 2017 | Ghana | 1082 women referred for high-risk delivery | 1 regional referral hospital | Level 4.c | 10-week descriptive study of incoming obstetric referrals, based on patient records and observations of waiting and triage times at arrival. | The analysis offers an overview of broad issues affecting the referral process, but with no breakdown by type of referring facility. The study identifies potential unnecessary referrals based on clinical condition of patient at arrival vs. diagnosis/reason for referral at sending institution, however appropriateness of referral not thoroughly assessed as it was not within the scope of the research. |
| Gyedu et al 2015 | Ghana | 643 incoming referrals for elective surgery | 1 tertiary referral hospital | Level 4.b | Quantitative study to assess deficiencies in essential referral information for incoming elective surgical cases, based on review of patient records for a 3-month period. | Descriptive study using clear and simple metrics to review and compare completeness of referral records received from other hospitals. The title does not reflect the content of the article, because the referral information is not assessed for quality (e.g. analysis of provisional diagnosis at sending institution vs. actual diagnosis at receiving hospital, appropriateness of pre-referral management etc. are missing). Also, no assessment of the effect of more complete forms vs more incomplete forms (e.g. triage, waiting times etc.). |
| Khan et al 2014 | Pakistan | 99 patients presenting with surgical emergencies, of which 49 transferred from another health facility and 50 self-referrals | 1 tertiary referral hospital | Level 3.c | Quantitative study assessing effects of delayed transfer of patients with surgical emergencies from other facilities, using direct arrivals as control group. Data collection over a 15-day period, using questionnaires and patients files. | The authors acknowledged the short duration and small sample size as limitations. Also, the investigation of relationship between transfer and clinical outcomes is based only on t-tests, limiting the depth of the analysis. |
| Lee 2008 | Malaysia | 65 infants with neonatal cholestasis referred from hospitals and primary health facilities | 1 tertiary hospital | Level 4.c | Prospective descriptive study of pre-admission consultations and reasons for delayed referral of infants with neonatal cholestasis. Based on review of referral letters, patients records, interviews with parents over a 3-year period. | This study included very small sample of patients, within one age-group and only one type of condition. Reasons for delayed referrals may be limited to this particular condition and patient profile. |
| Nkurunziza et al 2016 | Rwanda | 282 trauma patients recommended for referral | 3 public district hospitals | Level 4.c | Retrospective quantitative study of delays in outward referrals of patients presenting with injuries. Based on 12 months of data from admission registers, patients’ charts, ward registers and hospital monthly referral reports. | The small sample size is acknowledged by the authors as a limitation to the study. Also, the three hospitals included in the study are not typical, because are part of a programme sponsored by an international NGO to support the poorest patients during the referral process in the form of financial and logistical assistance, among other things. Some of the patients in this study might have benefitted from the programme, so the percentage of referral delays due to financial reasons might have been affected. |
| Simba et al 2008 | Tanzania | 12688 patients (of which 1936 referred from other public and private hospitals within Dar es Salaam). Referring clinicians (# not specified) | 1 tertiary referral hospital and 29 referring facilities (of which 3 public hospitals and 6 private hospitals) | Level 4.b | Descriptive study of incoming referral patterns based on prospective review of patients records over a 10-week period and interviews with referring clinicians. | While patients requiring surgical services made up a large part of the study population (66.8%), the study examined all referrals received at the hospital. Results, while informative about the functionality of the overall referral system, are not specific to surgical referrals from district hospitals. Additionally the geographical focus of the study is Dar es Salaam, one of the largest cities in Tanzania, so results are not entirely representative of the situation country-wide. The paper provides frequencies of reasons for referrals reported by sending facilities but no in-depth analysis is included. Statement about appropriateness of referral not backed up by study findings. Background information about interview respondents missing. |
| Siraj et al 2016 | Pakistan | 347 obstetric patients referred onward | 2 district hospitals | Level 4.b | Quantitative study to identify number and causes of obstetric referrals from district hospitals to tertiary care level over a 6-month period. Based on a bespoke referral register and review of referral letters. | Descriptive analysis weakened by inaccurate and misleading figures reported in sections of the manuscript (e.g. self-referrals grouped with referrals, mistakes in reported figures etc.). Some of the statements in the discussion not supported by scientific evidence gathered by the study. |
| Fleming et al 2017 | Nepal | 292 patients referred for outside surgical care. 10 clinical and operational staff. | 1 public district hospital | Level 4.d | Intervention study to evaluate a surgical referral and care coordination programme where financial, social and logistical support provided to patients throughout the referral process. Study based on chart reviews, cost data, interviews | Descriptive study done in one hospital only. Methodology for evaluating the effect of the intervention not clear, with no clearly defined objectives. Results are not presented in the relevant sections, but scattered across the discussion. |
| Rudge et al 2011 | Brazil | 27,387 deliveries | 1 Level 2 hospital and 1 Level 3 hospital | Level 4.b | Cross-sectional study of an intervention to facilitate exchange and treatment of obstetric patients between a Level 2 and Level 3 hospital based on assessment of clinical risk factors. The aim was to improve use of public resources and reduce overcrowding at the tertiary hospital. Study based on review of patients and hospital records, over a 12-year period. | Sound study, even if results somehow limited by absence of a control group and baseline data. It would have been interesting to assess the cost associate with the intervention to gather a deeper insight into the effectiveness of the programme. |
| Sani et al 2009 | Niger | Surgical patients managed by DH doctors who completed the new training programme (# no specified). | 1 regional hospital and 3 district hospitals | Level 4.b | Cross-sectional descriptive study evaluating a new training programme to enhance provision of surgical services at district hospitals. Based on review of patients charts, hospital records, regional reports, supervisors reports and qualitative. The study, running for 1 year, tracked multiple indicators, including the trends in no. of transfer of emergency cases from the district hospitals to the regional hospital. | Results should be interpreted with caution as surgical service provision at district level prior to the introduction of this new ministry-led initiative was minimal (mostly outreach programmes) and comparative data on this particular aspect not reported in the study. Only one region with one regional and four district hospitals included in the study. |
| Shi et al 2014 | China | 359 patients admitted for digestive tract cancer surgery at the tertiary hospital | 1 tertiary hospital and 3 secondary hospitals | Level 3.c | Retrospective controlled cohort study to examine the impact of elective referrals from outreach specialists on hospitalisation costs of rural patients requiring advance surgery at a tertiary hospital to treat digestive tract cancer. 2-year data based on patient and hospital records. Patients grouped as admitted to the tertiary hospital without referral, referred by local doctors at the sending hospitals or referred by the outreach specialists at the sending hospitals. | Data on management costs of the intervention not available for the study. |
