# Supplementary material for: Surgical referral systems in low- and middle-income countries: A review of the evidence
Source: PLoS One. 2019 Sep 27;14(9):e0223328. doi: 10.1371/journal.pone.0223328 (PMC6764741; doi:10.1371/journal.pone.0223328)
Supplement: Attachment — Submitted filename: Response to Reviewers.docx [file pone.0223328.s004.docx]

09 September 2019

Dear Editor,

we would like to take this opportunity to thank you and the Reviewers for the positive feedback on our manuscript and for the valuable comments and suggestions for improvement. As requested, please find below our responses and clarifications.

Best wishes on behalf of the authors,

Chiara Pittalis

**Comments:**

*1. Please state in your methods section the start and end date of searches.*

R: thank you for the advice, we have added the dates to the manuscript.

*1. Since tables must be stand alone and understandable, please explain what JBI means in the table “S2 Table. Characteristics of studies included in the review”.*

R: we have modified the table heading accordingly.

*2. Page 20, line 282, please check for typographical errors.*

R: thank you for spotting the typo, we have corrected it.

*3. Page 29, line 296, please check for typographical errors.*

R: we have corrected the typo accordingly.

*4. How is reference 28 as mentioned in text used in the evaluation of the quality of evidence? The table S2 suggests that JBI which is reference 27 was used. Please clarify this.*

R: reference no. 27 is the Thaddeus and Maine ‘three delays’ model, which informed the thematic analysis of the papers as described in the ‘Data charting and analysis’ section. We are assuming the confusion may be between the JBI tools and reference no. 28 – Atkins et al paper on how to grade quality of evidence and strength of recommendations. We used the JBI tools and methods to conduct an in-depth analysis of the reviewed papers, the outcomes of which are presented in the Supplementary Table.

The Atkins et al framework categorises type of evidence as high, low and very low, and it helped us summarise in a simple and systematic way our overall reflections on the strength of the studies being assessed and confidence placed in their recommendations. This is mentioned in lines 220-221, pg. 12 of our manuscript. Since all reviewed studies were observational and mostly descriptive in nature, the quality of evidence/recommendations was graded as low.

*5. How many and which papers are you referring to in this statement “some of them mention that surgery is not typically among the top priorities for local governments, with implications…… (page 20 line 280 -281)?’’*

R: in our review this statement derives from Albutt et al paper (we have modified lines 280-281 in the manuscript to clarify), but the issue of lack of financing for surgery in Africa is supported by the wider literature. One good report is the paper by Citron, Chokotho and Lavy published in 2016: ‘Prioritisation of surgery in the national health strategic plans of Africa: a systematic review’ (DOI:<https://doi.org/10.1016/S0140-6736(15)60848-0>).
6*. How many and which papers are you referring to in this statement ‘A number of studies pointed to the influence of wider country contexts……?’. Are the examples you cite in the text that follows all the papers referred to in this statement or are there more?*

R: yes, the six examples in our manuscript are the ones we referred to in that opening statement (line 290 onwards).

*7. For the table ‘S1 Table: Search string’, present the search string for the other databases besides MEDLINE which were also searched in order for readers to know and understand how the search strategy was adopted to these databases to facilitate reproducibility of your search.*

R: as requested, we have added all search strings to the Supplementary Table.
